# Electrospun encapsulation of grape pomace extract: *in vitro* antioxidant, anti‐inflammatory, and antihyperglycemic properties

**DOI:** 10.1002/jsfa.70506

**Published:** 2026-02-05

**Authors:** Estefani Tavares Jansen, Elder Pacheco da Cruz, Laura Martins Fonseca, Marjana Radünz, Taiane Mota Camargo, Alvaro Renato Guerra Dias, Elessandra da Rosa Zavareze

**Affiliations:** ^1^ Laboratory of Biopolymers and Nanotechnology in Food (BioNano), Graduate Program in Food Science and Technology, Department of Agroindustrial Science and Technology Federal University of Pelotas Pelotas Brazil; ^2^ Faculty of Nutrition Federal University of Pelotas Pelotas Brazil; ^3^ Department of Agroindustrial Science and Technology Federal University of Pelotas Pelotas Brazil

**Keywords:** agricultural by‐product, anthocyanins, α‐amylase, bioactivity, α‐glucosidase, ultrafine fibers

## Abstract

**BACKGROUND:**

The escalating prevalence of lifestyle‐ and aged‐related conditions, including diabetes, chronic inflammation, and cardiovascular disorders, underscores the urgent need for natural therapies. Such alternatives should offer reduced side effects compared to conventional pharmaceuticals while playing a proactive role in disease prevention. Red wine production generates grape pomace, a by‐product rich in valuable yet unstable anthocyanins. Encapsulation by electrospinning can protect these compounds. This study aimed to encapsulate grape pomace extract (5%, 10%, and 15%, *w/w*) into electrospun ultrafine zein fibers and to evaluate the *in vitro* biological activities, including antioxidant, antihyperglycemic, and anti‐inflammatory properties.

**RESULTS:**

Encapsulated grape pomace extract demonstrated antioxidant activity, showing the ability to capture hydroxyl and nitric oxide free radicals. It exhibited an antihyperglycemic effect by inhibiting carbohydrate‐digesting enzymes, α‐amylase, and α‐glucosidase. Anti‐inflammatory activity was observed through the inhibition of thermal protein denaturation. The evaluated activities were concentration‐dependent, with fibers containing 15% (*w/w*) extract showing the highest *in vitro* bioactivity.

**CONCLUSION:**

Our findings suggest that grape pomace extract encapsulated in ultrafine zein fibers is a promising new formulation for natural medicine. This potential extends to being integrated into functional foods, offering a dual application for health promotion and potential uses in food and pharmaceutical products. © 2026 The Author(s). *Journal of the Science of Food and Agriculture* published by John Wiley & Sons Ltd on behalf of Society of Chemical Industry.

## INTRODUCTION

The convergence of increased global life expectancy and shifts in behavioral patterns has led to a rise in the diagnosis of several diseases, including diabetes, cancer, and cardiovascular and chronic inflammatory diseases. This phenomenon is associated with elevated levels of oxidative stress and inflammation. Currently, the increase in the number of cases of these pathologies is considered a serious public health problem.^1^, ^2^

Type II diabetes mellitus is a major cause of premature death, where ineffective insulin use leads to hyperglycemia (a high concentration of glucose in the bloodstream). This triggers a self‐perpetuating cycle of oxidative stress, inflammation, and worsening insulin resistance.^3^, ^4^ While inflammation is a normal defense against injury and infections, its chronic persistence leads to systemic damage and is associated with serious conditions such as cancer, cardiovascular disease, neurodegenerative disorders (Alzheimer's and Parkinson's disease), asthma, and depression.^5^, ^6^, ^7^ Some of these conditions do not respond satisfactorily to existing conventional treatments, or these treatments have several undesirable side effects.^4^, ^8^, ^9^ Thus, there is an increasing interest in identifying new naturally occurring compounds that may provide health benefits by controlling oxidative stress, inflammation, and hyperglycemia.^3^, ^10^ Phenolic compounds are a standout group of these compounds, which can be sourced either from food or through nutritional supplements.^11^, ^12^

Anthocyanins are natural water‐soluble pigments from the group of phenolic compounds widely present in nature.^13^, ^14^ These molecules are recognized for their relevant biological activities, such as antioxidant,^15^ antimicrobial,^16^ antifungal,^17^ anti‐inflammatory,^1^ antihyperglycemic,^9^ and anticancer,^18^ among others. This demonstrates significant potential for application and economic value across the food, pharmaceutical, and cosmetics industries.^19^

Anthocyanins can be obtained from various natural sources.^20^ However, in recent years, efforts have been made to obtain these biomolecules from grape pomace. This solid waste is generated in large quantities every year during the production of juice and red wine and represents between 20% and 25% of the initial weight of the grapes.^21^, ^22^, ^23^ The valorization of this agricultural by‐product presents itself as an innovative alternative source for obtaining anthocyanins from grapes and brings environmental, economic, and social benefits. In addition, it is in line with consumers' growing concern with the consumption of products that generate less environmental impact.^24^, ^25^

Despite the benefits of using anthocyanins from grape pomace, the stability of these molecules is a critical factor, as they are easily degraded by external factors such as pH, light, temperature, oxygen, metal ions, and enzymes.^17^, ^18^ Therefore, it is crucial to use encapsulation techniques that can provide greater stability to them.^26^, ^27^, ^28^

Electrospinning is a simple, versatile, cost‐effective, room‐temperature method for nanoencapsulation that use biopolymers (including chitosan, cellulose, starch, whey protein isolate, and zein). It produces fibers with high porosity, large surface area, and high loading capacity compared to conventional techniques.^29^, ^30^, ^31^, ^32^

In the available literature, only one previous study has been identified on this topic, conducted by our own research group. In that initial work, the technique of nanoencapsulation by electrospinning was used to produce zein fibers capable of incorporating grape pomace extract, an agricultural by‐product generated after the production of Bordô red wine. The result was quite promising: smooth, cylindrical, and uniform fibers, without the presence of beads. The average diameter of fibers was approximately 450 nm (classifying them as ultrafine fibers), and a loading capacity of up to 82%. In addition, these fibers were able to thermally protect the encapsulated compounds and maintain their antimicrobial activity.^33^

Given these satisfactory results, we proceeded with the study by focusing on the biological activities of this material. We hypothesize that the encapsulation of Bordô grape pomace extract in ultrafine zein fibers may represent an innovative and promising alternative for applications in the food and pharmaceutical industries. Thus, the objective of this study was to evaluate the biological activities of grape pomace extract, both in its unencapsulated and encapsulated forms, focusing on three main aspects: (i) antioxidant activity against hydroxyl and nitric oxide free radicals; (ii) antihyperglycemic activity through the inhibition of the enzymes α‐amylase and α‐glucosidase; and (iii) anti‐inflammatory activity based on inhibition of protein denaturation by heat. Exploring the antidiabetic and anti‐inflammatory potential of encapsulated grape pomace extract is essential for addressing chronic metabolic diseases. This study pioneers the development of ultrafine zein fibers to evaluate these critical, yet previously unreported, therapeutic actions.

## MATERIALS AND METHODS

### Materials

The grape pomace extract was provided by a winery located in Morro Redondo (−31.6222 S; −52.7820 W; Rio Grande do Sul, Brazil). Ethanol p.a. (CAS 64‐17‐5) was purchased from Dynamic Chemical Contemporary Ltda. (Indaiatuba, São Paulo, Brazil) Hydrochloric acid p.a. (CAS 7647‐01‐0) and hydrogen peroxide p.a. were purchased from Synth. Zein (CAS 9010‐66‐6, Diadema, São Paulo, Brazil), pancreatic α‐amylase (CAS 8049‐47‐6), α‐glucosidase (CAS 9001‐42‐7), Griess reagent, and salicylic acid (CAS 69‐72‐7) were purchased from Sigma‐Aldrich (St Louis, MO, USA). Soluble starch (CAS 9005‐84‐9), *p*‐nitrophenyl‐*α*‐d‐glycopyranoside (CAS 3767‐28‐0), sodium nitroprusside (CAS 13755‐38‐9), phosphoric acid (CAS 7664‐38‐2), heptahydrate iron sulfate (CAS 7782‐63‐0), iodine (CAS 7553‐56‐2), and potassium iodide (CAS 7681‐11‐0) were obtained from Merck (Darmstadt, Germany). All other reagents used in the analyses were used in commercial form without prior purification. Ultrapure water was obtained by the MegaPurity® water purification system (MecLab, Brazil).

### Extraction and characterization of grape pomace extract

Bordô grape pomace (*Vitis labrusca* L.), obtained after red wine production, was composed of approximately 75% skins and pulp and 25% seeds. This material was stored in an ultra‐freezer (IULT‐86°; Indrel Scientific, Londrina, Paraná, Brazil), and after manual separation of the seeds, the grape pomace (fraction of skins and pulp) was lyophilized (Liotop K108; Liobras, São Carlos, São Paulo, Brazil) and ground in an analytical mill (OMDR‐100; Oster, USA) for 40 s at maximum speed.

The production of grape pomace extract was carried out in a two‐stage process, according to the methodology of Prietto *et al*.,^34^ with modifications. Initially, 30 g of grape pomace (freeze‐dried and ground) was added to a 75% ethanol (*v/v*, in water) solution acidified with hydrochloric acid (1%, *v/v*). This mixture was protected from light and was continuously stirred (RW‐20 digital; IKA, Staufen, Germany) for 2 h at 25 °C. After, the solution was centrifuged (RC5C; Sorvall Instruments, Newtown, USA) at 12 000 × *g* for 10 min, and Supernatant 1 was separated. In the second step, 600 mL of the ethanol solution (75%, *v/v*, in water) was added to the grape pomace mass, and the mixture was again agitated for 2 h. Centrifugation was then performed, and Supernatant 2 was collected. Supernatants 1 and 2 were homogenized. The grape pomace extract was concentrated in a rotary evaporator (Laborota 4000eco; Heidolph, Schwabach, Germany) for 20 min at 50 °C (ultrathermostatic bath Q214M2; Quimis, Brazil) and subsequently freeze‐dried (Liotop K108; Liobras). Finally, the extract was stored at −18 °C (CVU20; Consul, Brazil).

The characterization of grape pomace extract for the identification and quantification of anthocyanins was performed using high‐performance liquid chromatography–mass spectrometry (HPLC–MS), as described in our previous study.^33^ Nine different anthocyanins molecules were identified (in descending order of quantification): malvidin‐3‐(6‐coumaroyl)‐glucoside, petunidin‐3‐(6‐coumaroyl)‐glucoside, delphinidin‐3‐*O*‐*trans*‐*p*‐coumaroyl‐glucoside, malvidin‐3‐glucoside, malvidin‐3,5‐diglucoside, petunidin‐3‐*O*‐glucoside, malvidin‐3‐*O*‐acetylglucoside, and cyanidin‐3‐glucoside. These compounds had a concentration equal to 1.29 g kg^−1^ of lyophilized extract.^33^

### Grape pomace extract encapsulation by the electrospinning process

The polymeric solution preparation and the electrospinning process were performed according to the methodology of a previous study.^33^ The polymeric solution was produced with zein (30%, *w/v*) as wall material diluted in ethanol (80%, *v/v*, in water) and stirred (Multistirrer 6; Velp Scientifica, Usmate, Italy) for 1 h. After, the grape pomace extract (freeze‐dried) was added to the polymeric solution in concentrations of 0.045 mg, 0.090 mg, and 0.135 mg relative to the zein mass (0.9 mg) in the polymeric solution. The resulting solutions were designated as 5%, 10%, and 15% (*w/w*), respectively. These solutions were protected from light and were stirred for another 10 min to completely homogenize the extract in the zein solutions. A control solution was prepared without the extract (0%, *w/w*).

The grape pomace extract encapsulation into zein fibers was performed in a horizontal electrospinning station. The collector plate was covered with aluminum foil, and each solution was placed in a plastic syringe (3 mL) with a stainless steel needle (0.8 mm diameter). The ultrafine fibers were produced with the following parameters: feed flow rate of 0.6 mL h^−1^ in an electric pump (Model 100; KD Scientific, Holliston, Massachusetts, USA), electrical voltage of +20 kV with a high‐voltage power supply (FA + 30 kV; Faíscas, Porto Alegre, Rio Grande do Sul, Brazil), and distance from the tip of the needle to the collector of 20 cm. The electrospinning process was developed in an environment with controlled temperature (20 ± 2 °C) (EcoTurbo; Electrolux, Manaus, Brazil) and relative humidity (47 ± 2%) (Q831M20; Quimis).

The zein ultrafine fibers produced without extract had a cylindrical, smooth, continuous, and homogeneous morphology, without the presence of beads. The encapsulation of the extract did not affect the morphology of the fibers. The average diameters observed were equal to 450 nm, classifying them as ultrafine fibers (diameter > 100 nm). In addition, the fibers demonstrated high loading capacities for the three extract concentrations incorporated (zein ultrafine fibers with 0%, 5%, 10%, and 15% *w/w*), with values between 76.12% and 81.99%. These data are important for further use of the materials.^33^

### Antioxidant activity

The antioxidant activity of unencapsulated and encapsulated grape pomace extract (ultrafine zein fibers with 0%, 5%, 10%, and 15% *w/w*) was evaluated against nitric oxide and hydroxyl free radicals. For these analyses, a solution was initially prepared for each sample by diluting 15 mg of unencapsulated and encapsulated extract in 750 μL of 80% ethanol (*v/v*, in water).

The results were expressed as a percentage of inhibition of nitric oxide or hydroxyl radicals, according to Eqn (1), where ‘ABS_blank_’ is the absorbance of the blank (ethanol 80%, *v/v*, in water) and ‘ABS_sample_’ is the absorbance of each sample.

(1) $$\text{Free radical inhibition}\;\left(\%\right)=\frac{{\mathrm{ABS}}_{\text{blank}}-{\mathrm{ABS}}_{\text{sample}}\;}{{\mathrm{ABS}}_{\text{blank}}}\times \;100$$

#### Nitric oxide radical inhibition

The antioxidant capacity against the nitric oxide radical was determined according to the methodology of Vinholes *et al*.,^35^ with some modifications. Initially, 50 μL of each sample solution was added to 50 μL of sodium nitroprusside (20 mmol L^−1^), and the mixture was kept under direct light for 1 h at room temperature (20 ± 2 °C). Subsequently, 50 μL of 2% phosphoric acid and 50 μL of Griess reagent were added, and the medium was incubated in the dark for 10 min. Finally, the absorbance was measured on a spectrophotometer (SpectraMax 190; Molecular Devices, San Jose, CA, USA) at a wavelength of 562 nm.

#### Hydroxyl radical inhibition

The antioxidant capacity against the hydroxyl radical was determined according to the methodology adapted from Vinholes *et al*.^35^ For this analysis, 25 μL of each sample solution, 110 μL of 8 mmol L^−1^ heptahydrate iron(II) sulfate, 50 μL of 7.18 mmol L^−1^ hydrogen peroxide, and 74.2 μL of 3 mmol L^−1^ salicylic acid were mixed, shaken for 30 s, and incubated at 37 °C in a spectrophotometer (SpectraMax 190; Molecular Devices) for 30 min. Afterwards, the reading was performed at a wavelength of 515 nm.

### Antihyperglycemic activity

#### Inhibition of α‐amylase

The inhibition potential of the α‐amylase enzyme (from porcine pancreas; Sigma‐Aldrich) was evaluated initially by preparing each sample solution with 15 mg of unencapsulated and encapsulated grape pomace extract (ultrafine zein fibers with 0%, 5%, 10%, and 15%, *w/w*) and 750 μL of 80% ethanol (*v/v*, in water). Then, 50 μL of pancreatic α‐amylase (241.71 U mL^−1^) and 200 μL of phosphate buffer (pH 7.0) were added to 60 μL of each sample solution, which were incubated at 37 °C for 5 min. Subsequently, 250 μL of soluble starch (4 mg mL^−1^) was added to the sample, which was kept at the same temperature for another 15 min. To stop the reaction, 50 μL of hydrochloric acid (1 mol L^−1^) was used. After, 100 μL of iodine solution (0.005 mol L^−1^) with potassium iodide (0.005 mol L^−1^) was added. The absorbance was measured in a spectrophotometer (SpectraMax 190; Molecular Devices) at 690 nm. The results were expressed as a percentage of inhibition of α‐amylase enzyme according to Eqn (2), where ‘ABS_blank_’ is the absorbance of the blank (ethanol 80%, *v/v*, in water) and ‘ABS_sample_’ is the absorbance of each sample.^4^

(2) $$\text{Enzyme}\;\text{inhibition}\;\left(\%\right)=\frac{{\mathrm{ABS}}_{\text{blank}}-{\mathrm{ABS}}_{\text{sample}}\;}{{\mathrm{ABS}}_{\text{blank}}}\;\times \;100$$

#### Inhibition of α‐glucosidase

The inhibition potential of the enzyme α‐glucosidase (from *Saccharomyces cerevisiae*; Sigma‐Aldrich) was evaluated adding each sample solution (prepared as described in Inhibition of α‐amylase section) (10 μL) to 50 μL of 3.25 mmol L^−1^ *p*‐nitrophenyl‐*α‐* d‐glycopyranoside substrate (diluted in pH 7.0 phosphate buffer) and 50 μL of enzyme (9.37 U mL^−1^ diluted in pH 7.0 phosphate buffer), and the mixture was kept at 37 °C for 10 min. After, the absorbance was measured in a spectrophotometer (SpectraMax 190; Molecular Devices) at 405 nm. The results were expressed as a percentage of inhibition of α‐glucosidase enzyme according to Eqn (2).^4^

### Anti‐inflammatory activity

To evaluate the anti‐inflammatory activity, initially, each sample solution was prepared by diluting 15 mg of unencapsulated and encapsulated grape pomace extract (ultrafine zein fibers with 0%, 5%, 10%, and 15%, *w/w*) in 750 μL of 80% ethanol (*v/v* in water). Afterwards, the reaction medium was formed by 120 μL of each sample solution, 12 μL of egg albumin (from fresh chicken egg), and 167 μL of phosphate‐buffered saline (PBS, pH 6.4). The mixture was maintained at 37 °C for 5 min and then heated to 70 °C for 5 min. After cooling, the absorbance was read at 660 nm in a spectrophotometer (SpectraMax 190; Molecular Devices). Diclofenac potassium (50 mg mL^−1^) was used as the reference drug and treated similarly to determine absorbance. The results were expressed as a percentage of protein denaturation (%), according to Eqn (3), where ‘ABS_sample_’ is the absorbance of each sample and ‘ABS_blank_’ is the absorbance of the blank (water).^36^

(3) $$\text{Protein denaturation}\;\left(\%\right)=\frac{{\mathrm{ABS}}_{\text{sample}}\;}{{\mathrm{ABS}}_{\text{blank}}-1}\;\times \;100$$

### Statistical analysis

The analysis was performed in triplicate. Statistical analysis of data was performed using analysis of variance (ANOVA) and comparison of means using Tukey's test (*p* < 0.05) (Statistica; StatSoft, Hamburg, Germany).

## RESULTS AND DISCUSSION

### Antioxidant activity

The antioxidant activity of the unencapsulated grape pomace extract was the highest among all samples (*p* < 0.05), with inhibitions of 45.36% and 59.13% for hydroxyl and nitric oxide radicals, respectively (Table 1). The difference observed in the results is possibly due to the greater interaction capacity of the anthocyanins present in the extract with nitric oxide radicals compared to hydroxyl radicals, which allows higher antioxidant activity against nitric oxide.

**Table 1:** Antioxidant activities of unencapsulated grape pomace extract and encapsulated in ultrafine zein fibers with 0%, 5%, 10% and 15%, *w/w*

Material	Extract concentration^†^ (%, *w/w*)	Radical inhibition (%)	
		Hydroxyl free radical	Nitric oxide free radical
Unencapsulated extract		45.36 ± 3.07^a^	59.13 ± 0.64^a^
Fibers 0%	0	ND	3.94 ± 2.18^d^
Fibers 5%	5	8.13 ± 1.81^c^	23.45 ± 1.22^c^
Fibers 10%	10	11.96 ± 2.90^c^	33.13 ± 3.75^b^
Fibers 15%	15	20.13 ± 1.71^b^	36.76 ± 3.77^b^

*Note*: Mean ± standard deviation (*n* = 3) followed by different letters in the same column indicate a significant difference between data by the Tukey test (*p* < 0.05). ND, not detected.

^†^ Concentration of encapsulated extract in ultrafine zein fibers expressed as a percentage (*w/w*, in relation to zein in polymeric solution).

The antioxidant activity of the encapsulated extract increased with its concentration, a finding consistent for both radical assays. When tested against the hydroxyl radical, fibers with 15% *w/w* extract showed the highest antioxidant activity, inhibiting 20.13%. The fibers with 10% and 5% extract showed no significant difference. For the nitric oxide radical, fibers with 15% (33.76%) and 10% (33.13%) *w/w* extract demonstrated similar antioxidant capacity, outperforming the 5% extract, which inhibited approximately 23% of the radicals.

The antioxidant activity of the unencapsulated and encapsulated extract can be explained by its characteristic molecular structure of phenolic compounds, such as anthocyanins. These molecules have one or more aromatic rings in a conjugated system and one or more hydroxyl groups. Thus, they are capable of donating an electron or a hydrogen atom to the free radical, such as hydroxyl and nitric oxide radicals, transforming it into a neutral molecule, without the formation of a new radical species.^12^

According to Brennan *et al*.,^37^ the neutralization of reactive oxygen species, such as hydroxyl, by anthocyanins can mitigate oxidative stress, a key factor in chronic diseases such as cancer and cardiovascular disease. In addition, the reduction in nitric oxide radical levels by anthocyanins is also important, as it is a pro‐inflammatory marker responsible for the formation of exudate, a sign of inflammatory reaction. This exudate is produced through the leakage of fluid from the capillaries into the damaged tissue as a result of vascular relaxation caused by nitric oxide.^1^

Furthermore, fibers without the extract (0% *w/w*, composed only of zein biopolymer) showed antioxidant activity against nitric oxide radicals. The effect on the free radical is likely due to the presence of amino acids in the zein chain that exhibit antioxidant activity both in free form and as residues in the protein structure.^38^, ^39^

Cruz *et al*.^29^ encapsulated lemongrass essential oil into cassava starch fibers and reported similar results for nitric oxide radical inhibition. The unencapsulated essential oil inhibited 62.5% of the radicals dispersed in the reaction medium, and the fibers with 0 to 40% oil inhibited 0.7% to 37.2%. However, concerning the hydroxyl radical, this same study reported better inhibition percentages, with 65.4% for the unencapsulated essential oil and between 2.4% and 46.6% for the fibers with 0 and 40% encapsulated oil, respectively.

Radünz *et al*.^40^ produced capsules based on the same biopolymer used in the present study (zein) and encapsulated a hydroalcoholic extract of broccoli. The unencapsulated extract showed a 45.3% inhibition of hydroxyl free radicals, similar to the unencapsulated grape pomace extract. However, the capsules with 50% broccoli extract captured only 5.4% of hydroxyl radicals, a result lower than the inhibition percentages observed for the three concentrations of grape pomace extract encapsulated in zein ultrafine fibers (the lowest inhibition equal to 8.13% in fibers with 5% *w/w* extract). Regarding the free radical nitric oxide, both in unencapsulated and encapsulated form, broccoli extract did not show antioxidant activity.

The electrospinning encapsulation technique successfully preserved the antioxidant activity of the grape pomace extract. This was evidenced by its continued, though reduced, inhibition of nitric oxide and hydroxyl free radicals compared to the unencapsulated extract. Thus, the data demonstrate the potential of this technique to promote greater protection and stability for the bioactive molecules present in the grape pomace extract, such as anthocyanins, while preserving their biological activity. However, the free extract could be easily degraded by environmental conditions, such as changes in pH, light, and temperature, since it is not protected by the wall material of the polymeric structure formed by the zein fibers. This behavior was reported in our previous study, in which, when the free extract was subjected to 100 °C, only approximately 27% of the identified anthocyanins were not completely degraded. However, when the extract was encapsulated at 15% *w/w* in the fibers, 43% of the identified anthocyanins were preserved, representing an approximate 59% increase in protection, demonstrating the need for encapsulation.

Assessing antioxidant activity against hydroxyl and nitric oxide free radicals is extremely important. The hydroxyl radical is the most important reactive oxygen species and can be produced in the human body, causing lipid peroxidation of membrane lipids, as well as other biological damage to the body. Nitric oxide is a reactive nitrogen species and an important marker of inflammatory processes. When present in high concentrations, it can also cause damage to the body, such as neuroinflammatory diseases.^41^, ^42^, ^43^ Thus, the *in vitro* inhibition for fibers with grape pomace extract of both radicals, even at low percentages, can benefit and protect the human body from biological damage.

In addition, zein fibers with grape pomace extract have already demonstrated the ability to act against the synthetic radicals DPPH and ABTS.^33^ Although assays with these radicals are widely used to estimate the chemical antioxidant capacity of bioactive compounds, these radicals are artificially synthesized and do not directly reflect physiological conditions. In contrast, reactive species such as hydroxyl and nitric oxide free radicals are generated endogenously in the body and are therefore more biologically relevant for assessing antioxidant potential, although they still represent simplified models of the *in vivo* environment.

This set of data demonstrates the *in vitro* potential antioxidant property of the material against different species of free radicals. However, this antioxidant action should be better elucidated in *in vivo* models and bioavailability studies, but it represents a starting point for future applications of zein fibers and grape pomace extract in the food, pharmaceutical, and cosmetic industries.

### Antihyperglycemic activity

Unencapsulated grape pomace extract showed the highest antihyperglycemic activity in terms of inhibition of α‐amylase and α‐glucosidase enzymes (*p* < 0.05), inhibiting approximately 88% and 90% of the enzymes, respectively (Table 2). The inhibition of enzymes by grape pomace extract was possibly observed due to the presence of anthocyanins in the extract's composition, since these biomolecules have the ability to inhibit α‐amylase and α‐glucosidase enzymes.^44^, ^45^ According to Mojica *et al*.^46^ and Visvanathan *et al*.,^47^ polyphenolic compounds, such as anthocyanins, can bind directly to α‐amylase and α‐glucosidase and/or form complexes with starch through hydrogen bonding and hydrophobic interactions, acting to inhibit these enzymes.

**Table 2:** Antihyperglycemic activity of unencapsulated grape pomace extract and encapsulated in ultrafine zein fibers with 0%, 5%, 10% and 15%, *w/w*

Material	Extract concentration^†^ (%, *w/w*)	Inhibition of enzyme (%)	
		α‐Amylase	α‐Glucosidase
Unencapsulated extract		88.33 ± 2.07^a^	90.14 ± 0.10^a^
Fibers 0%	0	ND	ND
Fibers 5%	5	28.18 ± 3.94^d^	19.07 ± 1.16^d^
Fibers 10%	10	49.35 ± 1.68^c^	22.24 ± 0.11^c^
Fibers 15%	15	71.92 ± 5.06^b^	26.98 ± 1.17^b^

*Note*: Mean ± standard deviation (*n* = 3) followed by different letters in the same column indicate a significant difference between data by the Tukey test (*p* < 0.05). ND, not detected.

^†^ Concentration of encapsulated extract in ultrafine zein fibers expressed as a percentage (*w/w*, in relation to zein in polymeric solution).

Regarding the encapsulated grape pomace extract, antihyperglycemic activity was dependent on the concentration of extract incorporated into the polymeric material (*p* < 0.05), similar to the behavior observed for antioxidant activity. For the α‐amylase enzyme, fibers with 15% *w/w* extract showed inhibition equal to 71.92%, while fibers with 5% inhibited less than half of this percentage (28.18%). For α‐glucosidase, the inhibition percentages ranged from approximately 27% to 19% for fibers with 15% to 5% *w/w* extract, respectively. Fibers without extract were unable to inhibit any of the enzymes.

It was also observed that the encapsulated extract showed different inhibition capacities against α‐amylase and α‐glucosidase. The inhibition of α‐amylase was superior to that of α‐glucosidase. This behavior can be explained by the fact that there are fewer interactions between the extract and α‐glucosidase when it is incorporated into the polymeric structure of zein ultrafine fibers. Since, for the unencapsulated extract, the inhibition of both enzymes was similar.

Ji *et al*.^48^ evaluated the *in vitro* inhibition of α‐amylase and α‐glucosidase enzymes using an anthocyanin‐rich bilberry extract and reported half‐maximal inhibitory concentration (IC_50_) values of 4.06 ± 0.12 mg mL^−1^ for α‐amylase and 0.31 ± 0.02 mg mL^−1^ for α‐glucosidase, respectively. Similarly, Yang *et al*.^9^ investigated the inhibitory activity of anthocyanins from blueberries and purple sweet potatoes against the same carbohydrate digestion enzymes (α‐amylase and α‐glucosidase). In this study, diacylated anthocyanins extracted from purple sweet potatoes showed the most pronounced inhibitory effect among the extracts evaluated for both α‐amylase (IC_50_ equal to 0.078 mg mL^−1^) and α‐glucosidase (IC_50_ equal to 1.56 mg mL^−1^).

In both of the earlier‐mentioned studies, the inhibition of α‐amylase and α‐glucosidase enzymes was concentration‐dependent, which is consistent with the results observed in the present study. These *in vitro* findings suggest a potential antihyperglycemic effect of anthocyanins, associated with the inhibition of enzymes involved in the digestion of carbohydrates.

Hyperglycemia has become a growing health problem in the last decade, which can cause diabetes and several other complications, such as blindness, heart attacks, and kidney failure. It is estimated that by 2030, approximately 643 million people will have diabetes. In 90% of cases, this disease presents as diabetes mellitus type II (insulin resistance), which is a chronic metabolic disorder that can affect different organs of the human body, in addition to causing comorbidities and more serious diseases.^49^, ^50^

The enzymes α‐amylase and α‐glucosidase are related to the onset of diabetes mellitus type II, as they influence the metabolism of glycocompounds. In the intestine, pancreatic α‐amylase completes the lysis of carbohydrates into disaccharides and short oligosaccharides, after the hydrolytic action initiated by salivary α‐amylase. α‐glucosidase acts on the small intestine mucosa in the last stage of carbohydrate digestion, converting disaccharides into monosaccharides, such as glucose, which are quickly absorbed into the bloodstream. Thus, the inhibition of these enzymes influences the breakdown and absorption of carbohydrates and causes a reduction in postprandial glucose levels, minimizing the effects of hyperglycemia. However, currently, traditional drugs used to achieve this inhibition cause several unwanted side effects in patients, such as abdominal distension, flatulence, and gastrointestinal disorders.^51^, ^52^, ^53^

In this context, the search for active compounds capable of inhibiting α‐amylase and α‐glucosidase enzymes safely and with fewer side effects has been the focus of research aimed at assisting in the treatment of diseases related to hyperglycemia. Furthermore, total inhibition of α‐amylase and α‐glucosidase enzymes is not desirable, as it could cause other health problems related to nutrient deficiency in the body, the emergence of problems associated with excess glucose not absorbed in the gastrointestinal tract, and the presence of undigested starch in the large intestine, which can be fermented by bacteria of intestinal flora.^4^, ^52^, ^54^

Incomplete enzyme inhibition is most appropriate for clinical treatment. The partial inhibition presented by grape pomace extract is beneficial and consistent with the expected effects on α‐amylase and α‐glucosidase enzymes. Thus, the extract can contribute to controlling postprandial glucose levels and act in the treatment of diabetes mellitus type II without promoting the unwanted harmful effects of total inhibition of the two enzymes.

Grape pomace extract, rich in anthocyanins, has demonstrated *in vitro* potential to act as an inhibitor of key enzymes in carbohydrate digestion. Furthermore, after encapsulation by electrospinning, zein ultrafine fibers can act to provide better protection for the bioactive molecules in the extract against adverse environmental conditions, such as pH changes in the stomach, allowing a higher concentration of these biomolecules to reach the intestine in an active state and exert their antihyperglycemic potential.

The results presented here demonstrated *in vitro* inhibition of α‐amylase and α‐glucosidase enzymes and potential use for hyperglycemia control. This set of data provides a starting point for the development of new studies with *in vivo* models that aim to evaluate in detail the effects of the use of this material produced with electrospinning in controlling the activity of α‐amylase and α‐glucosidase, allowing the development of a future safe and natural alternative for the control and/or prevention of diabetes mellitus type II.

### Anti‐inflammatory activity

The *in vitro* anti‐inflammatory activity expressed in terms of inhibition of egg albumin protein denaturation by heat is shown in Table 3. The unencapsulated grape pomace extract and encapsulated at 15% *w/w* showed anti‐inflammatory activity comparable to that of diclofenac potassium (*p* < 0.05), a reference drug used in the treatment of inflammatory processes.

**Table 3:** Anti‐inflammatory activity of unencapsulated grape pomace extract and encapsulated in ultrafine zein fibers with 0%, 5%, 10% and 15%, *w/w*

Material	Extract concentration^†^ (%, *w/w*)	Inhibition of thermal protein denaturation (%)
Nonencapsulated extract		62.05 ± 2.43^a^
Fibers 0%	0	19.51 ± 2.77^d^
Fibers 5%	5	37.10 ± 0.36^c^
Fibers 10%	10	46.83 ± 0.21^b^
Fibers 15%	15	59.19 ± 1.53^a^
Diclofenac potassium		59.63 ± 3.35^a^

*Note*: Mean ± standard deviation (*n* = 3) followed by different letters in the same column indicate a significant difference between data by the Tukey test (*p* < 0.05).

^†^ Concentration of encapsulated extract in ultrafine zein fibers expressed as a percentage (*w/w*, in relation to zein in polymeric solution).

The ability to inhibit protein denaturation was dependent on the concentration of encapsulated extract (*p* < 0.05), decreasing from approximately 59% to 37% when the concentration of encapsulated extract was reduced from 15 to 5% *w/w*.

Protein denaturation is a process that can be caused by different external agents, such as the presence of acids, bases, and solvents, as well as heat. According to Kola *et al*.,^55^ protein denaturation is a fundamental cause of inflammation in the human body; consequently, *in vitro* assays serve as a vital screening tool for identifying novel agents with anti‐inflammatory properties. There are mentions in the literature showing that the production of autoantigens in certain arthritic diseases may be associated with protein denaturation *in vivo*. Thus, compounds capable of interfering with this process can be considered, in an exploratory manner, as potential agents with anti‐inflammatory activity.^55^

In this denaturation process, molecules suffer structural changes due to alterations in the interactions that maintain the secondary and tertiary structures of the protein, such as hydrogen bonds and hydrophobic, electrostatic, and disulfide interactions, losing their characteristic structural conformation. These changes usually cause the loss of the protein's biological activity and contribute to the production of autoantigens, leading to a series of autoimmune dysfunctions, such as inflammatory and rheumatoid disorders. This condition is usually observed in chronic inflammatory diseases associated with the immune system and age, such as neurodegenerative disorders, rheumatoid arthritis, atherosclerosis, and lupus erythematosus.^5^, ^8^, ^36^, ^56^ Thus, while inflammation is beneficial to human health and maintains the body safe from infections and injuries. Chronic inflammation needs to be properly controlled to prevent the chronic inflammatory process from causing damage to the organism and progressing to the diseases mentioned earlier.

The data reported here demonstrate the anti‐inflammatory *in vitro* effect based on inhibiting thermal protein denaturation of grape pomace extract, similar to a recognized drug. However, because it is of natural origin, this compound may have fewer side effects. Standard anti‐inflammatory drugs available on the market are often associated with unpleasant reactions, such as gastric irritation and ulcers. Electrospinning encapsulation did not negatively affect the anti‐inflammatory activity of the extract, and the polymeric structure formed by the zein ultrafine fibers provided greater protection to the encapsulated extract when compared to its unencapsulated form, as already discussed in previous sections. Therefore, zein fibers and grape pomace extract have the potential to act as a future natural alternative for inhibiting protein denaturation by heat, preserving their three‐dimensional structures, and acting as an anti‐inflammatory agent. However, *in vivo* applications and clinical studies need to be developed for a better understanding of the anti‐inflammatory properties of the material for applications in the food and/or pharmaceutical sectors.

Kola *et al*.^55^ used a similar method based on inhibiting the thermal denaturation of chicken egg protein to evaluate four hydroalcoholic extracts from natural African plants (roots of *Cochlospermum planchonii*, leaves of *Piliostigma thonningii*, leaves of *Paullinia pinnata*, and roots of *Securidaca longipedunculata*) in comparison with the reference drugs diclofenac and aspirin. The authors reported that all extracts showed anti‐denaturing potential, with IC_50_ ranging from 30.77 to 55.11 μg mL^−1^. It is noteworthy that the roots of *Securidaca longipedunculata* present an IC_50_ (30.77 ± 1.77 μg mL^−1^) lower than that observed for both drugs (IC_50_ diclofenac sodium and aspirin equal to 37.90 ± 1.62 and 41.68 ± 1.09 μg mL^−1^, respectively) demonstrating, under *in vitro* conditions, the potential of these roots for the development of agents with anti‐inflammatory effects associated with the inhibition of protein denaturation by heat.

Chandra *et al*.^36^ also evaluated the inhibition of chicken egg protein denaturation *in vitro* using an aqueous extract of coffee bean (*Coffea arabica* Linn. Rubiaceae) with diclofenac sodium as a positive control. The authors observed that the anti‐inflammatory effect, expressed by the inhibition of thermal protein denaturation, was dependent on the concentration of the extract. Similar behavior was observed in the present study, in which the anti‐denaturing activity of the fibers was shown to be dependent on the concentration of the extract incorporated into zein fibers. Furthermore, the coffee extract had an IC_50_ (40 μg mL^−1^) approximately 15 times lower than that of the standard drug (diclofenac sodium = 625 μg mL^−1^), suggesting high efficacy *in vitro*.

In relation to anthocyanins, there are several reports in the literature on the anti‐inflammatory effect of this molecules, which were evaluated in isolated form, such as pelargonidin‐3‐*O*–glycoside^1^ and malvidin‐3‐*O‐β*‐glucoside,^57^ or in extracts obtained from different botanical sources, such as strawberry,^1^ blackberry, elderberry,^2^ Polana raspberry,^12^ black raspberry,^58^ and wild blueberry.^59^ However, no study has yet been reported that evaluates the anti‐inflammatory biological activity for inhibiting thermal protein denaturation by anthocyanin‐rich extract produced from the winemaking process and incorporated into ultrafine zein fibers by electrospinning.

Fibers with 15% *w/w* extract showed the most promising results for the three biological activities evaluated *in vitro*: antioxidant, antihyperglycemic, and anti‐inflammatory. This performance was probably associated with the higher concentration of extract incorporated in relation to the other formulations (5% and 10% *w/w*), as all activities were dependent on the concentration of grape pomace extract. These outcomes indicate the potential of encapsulating the extract at a higher concentration (15%, *w/w*) in biodegradable sources, such as corn protein (zein) for the development of bioactive systems. While these findings are promising, the application of the fibers as a natural medicine or functional foods remains prospective, pending rigorous *in vivo* validation to confirm the observed health benefits. Thus, this study establishes a partial exploratory foundation for the biological activities of zein fibers containing grape pomace extract. Future research should prioritize *in vivo* models, bioavailability assessments, and scale‐up feasibility to fully characterize the material's properties for industrial application in food and pharmaceuticals.

In addition, zein fibers with grape pomace extract showed antimicrobial activity against Gram‐positive and Gram‐negative strains (as previously demonstrated in Jansen *et al*.^33^) and may also act to preserve and extend the shelf life of the functional food to which they are added. This makes it possible to reduce or even replace the use of synthetic additives for product conservation. This change is not only beneficial to consumer health, as it reduces the use of chemical preservatives, but is also in line with new consumer preferences for the manufacture of ‘clean label’ products.

Moreover, fibers with 15% *w/w* grape pomace extract showed a controlled release profile in simulated hydrophilic medium (10% ethanol, *v/v*, in water) for the different anthocyanins that compose the extract. According to our previous study,^33^ malvidin‐3,5‐diglucoside showed maximum release in 3 h, while other anthocyanin molecules, such as petunidin‐3‐*O*‐glucoside, malvidin‐3‐glucoside, delphinidin‐3‐*O‐trans‐p‐*coumaroyl‐glucoside, malvidin‐3‐(6‐coumaryl)‐glucoside and, petunidin‐3‐(6‐coumaryl)‐glucoside were released in higher concentrations over 48 h. This controlled release profile observed for the different anthocyanins was possibly influenced by the different types of interactions between each anthocyanin molecule and the zein polymer matrix, and the occurrence of the diffusion phenomenon.

The controlled release profile gives zein fibers with 15% *w/w* extract offers a versatile application potential, particularly as an active packaging component to preserve food quality and extend shelf life. In addition, the controlled release observed in a hydrophilic environment suggests the possibility of gradual release of anthocyanins throughout the human digestive system due to the presence of water inherent in this process, due to the presence of saliva and other digestive fluids. However, *in vitro* gastrointestinal digestion simulations are essential to evaluate the release kinetics of anthocyanins from zein fibers following ingestion, whether consumed as a natural medicine or as an active ingredient in functional foods.

The lower concentrations of extract incorporated into the fibers associated with the gradual release profile of anthocyanins may be related to the reduction in biological activities observed for antioxidant and antihyperglycemic properties when compared to the unencapsulated extract (Tables 1 and 2). The process of anthocyanin diffusion from the zein fibers matrix may be in its early stages. This may limit the interactions between these bioactive molecules and their respective targets in each assay – hydroxyl and nitric oxide free radicals or α‐amylase and α‐glucosidase enzymes, respectively. This behavior possibly explains the lower percentages of antioxidant and antihyperglycemic activity observed for zein fibers containing grape pomace extract, compared to the greater results of the free extract (unencapsulated). However, as previously discussed, the unencapsulated extract is more susceptible to degradation by environmental factors and can be rapidly consumed, since it is fully exposed to the environment and does not have a controlled release profile.

## CONCLUSIONS

Grape pomace extract encapsulated in zein ultrafine fibers showed significant biological activity *in vitro*, being able to sequester hydroxyl and nitric oxide free radicals. It also demonstrated antihyperglycemic activity in terms of inhibiting carbohydrate‐digesting enzymes, the α‐amylase and α‐glucosidase. Furthermore, the encapsulated extract showed anti‐inflammatory effects, inhibiting thermally induced denaturation of egg proteins. All these biological activities were dependent on the concentration of extract incorporated into the fibers. In addition, the results demonstrate that the encapsulation process maintains the biological activities of the unencapsulated extract.

These findings support the potential application of this material in the development of functional foods or natural medicines aimed at preventing and treating oxidative stress, diabetes, and chronic inflammation. Furthermore, the use of an anthocyanin‐rich extract obtained from grape pomace simultaneously contributes to reducing the volume of residue generated by the wine and juice industries and adding value to this agroindustrial by‐product, thereby contributing to the development of a circular economy.

For future studies, the ideal concentration of zein fibers containing the encapsulated extract should be investigated in order to understand the optimal range of biological activity and avoid both saturation effects and insufficient levels to promote significant biological responses. In addition, the beneficial effects already observed in *in vitro* assays should be investigated in *in vivo* models, covering the entire process during gastrointestinal tract digestion. Such evaluations are essential to determine the bioaccessibility and bioavailability of encapsulated grape pomace extract and to confirm whether its biological activity is preserved throughout the digestion process. Furthermore, evaluating its *in vivo* performance is crucial to ensuring the efficacy and safety of grape pomace extract encapsulated in ultrafine zein fibers in the food and pharmaceutical industries.

## CONFLICT OF INTEREST

The authors declare no conflict of interest.

## Data Availability

All relevant data are within the manuscript and available from the corresponding author upon request.
